# Strengthening Referral Networks for Management of Hypertension Across the Health System (STRENGTHS) in western Kenya: a study protocol of a cluster randomized trial

**DOI:** 10.1186/s13063-019-3661-4

**Published:** 2019-09-09

**Authors:** Tim Mercer, Benson Njuguna, Gerald S. Bloomfield, Jonathan Dick, Eric Finkelstein, Jemima Kamano, Ann Mwangi, Violet Naanyu, Sonak D. Pastakia, Thomas W. Valente, Rajesh Vedanthan, Constantine Akwanalo

**Affiliations:** 1 The University of Texas at Austin Dell Medical School, 1701 Trinity St., Austin, TX 78712 USA; 2 Moi Teaching and Referral Hospital, PO Box 3-30100, Eldoret, Kenya; 3 Duke University School of Medicine, Duke Clinical Research Institute and Duke Global Health Institute, 2301 Erwin Rd., Durham, NC 27704 USA; 4 Indiana University School of Medicine, 535 Barnhill Dr., Indianapolis, IN 46202 USA; 5 Duke-NUS Medical School, Singapore, 8 College Road, Singapore, 169857 Singapore; 6 Moi University School of Medicine, PO Box 4606, Eldoret, 30100 Kenya; 7 Purdue University College of Pharmacy, 575 Stadium Mall Dr., West Lafayette, IN 47907 USA; 8 Keck School of Medicine University of Southern California, 2001 N Soto Street, Soto Street Building, Suite 330, MC 9239, Los Angeles, CA 90089-9239 USA; 9 New York University School of Medicine, 180 Madison Avenue, 8th Floor, New York, NY 10016 USA

**Keywords:** Referral networks, Hypertension, Cardiovascular disease, Health systems, Health systems strengthening, Implementation science, Health information technology, Peer support, Low- and middle-income countries (LMICs), Kenya

## Abstract

**Background:**

Hypertension is a major risk factor for cardiovascular disease (CVD), yet treatment and control rates for hypertension are very low in low- and middle-income countries (LMICs). Lack of effective referral networks between different levels of the health system is one factor that threatens the ability to achieve adequate blood pressure control and prevent CVD-related morbidity. Health information technology and peer support are two strategies that have improved care coordination and clinical outcomes for other disease entities in other settings; however, their effectiveness and cost-effectiveness in strengthening referral networks to improve blood pressure control and reduce CVD risk in low-resource settings are unknown.

**Methods/design:**

We will use the PRECEDE-PROCEED framework to conduct transdisciplinary implementation research, focused on strengthening referral networks for hypertension in western Kenya. We will conduct a baseline needs and contextual assessment using a mixed-methods approach, in order to inform a participatory, community-based design process to fully develop a contextually and culturally appropriate intervention model that combines health information technology and peer support. Subsequently, we will conduct a two-arm cluster randomized trial comparing 1) usual care for referrals vs 2) referral networks strengthened with our intervention. The primary outcome will be one-year change in systolic blood pressure. The key secondary clinical outcome will be CVD risk reduction, and the key secondary implementation outcomes will include referral process metrics such as referral appropriateness and completion rates. We will conduct a mediation analysis to evaluate the influence of changes in referral network characteristics on intervention outcomes, a moderation analysis to evaluate the influence of baseline referral network characteristics on the effectiveness of the intervention, as well as a process evaluation using the Saunders framework. Finally, we will analyze the incremental cost-effectiveness of the intervention relative to usual care, in terms of costs per unit decrease in systolic blood pressure, per percentage change in CVD risk score, and per disability-adjusted life year saved.

**Discussion:**

This study will provide evidence for the implementation of innovative strategies for strengthening referral networks to improve hypertension control in LMICs. If effective, it has the potential to be a scalable model for health systems strengthening in other low-resource settings worldwide.

**Trial registration:**

Clinicaltrials.gov, NCT03543787. Registered on 29 June 2018.

**Electronic supplementary material:**

The online version of this article (10.1186/s13063-019-3661-4) contains supplementary material, which is available to authorized users.

## Background

Elevated blood pressure (BP) is a leading preventable cause of cardiovascular disease (CVD) and premature death and disability globally [1, 2]. Hypertension treatment and control rates are low worldwide, but worst in low- and middle-income countries (LMICs), where less than 37% of patients are on treatment and 13% have adequately controlled BP [3]. Interventions to strengthen LMIC health systems to control hypertension are urgently needed [4].

Most LMICs utilize a tiered health care system, spanning a primary, secondary, and tertiary care continuum, with the goal of broadening access to basic health services through decentralizing, and decongesting higher-level facilities [5, 6]. The tiers are connected by referral networks which enable triaged access to different levels of care depending on patients’ needs. The effectiveness of these networks, however, is often inadequate, characterized by suboptimal rates of referral completion [7–10]. Key barriers to successful referral completion include logistical issues, cost, knowledge gaps among providers, lack of communication across levels of care, and inability to track referred patients [8, 11–15]. Successful referral completion has been shown to improve CVD care in high-income countries [16, 17] as well as HIV care in LMICs [18, 19]. However, studies evaluating interventions to improve referral networks for hypertension in LMICs are lacking.

Health information technology (HIT) and peer support target many of the barriers to successful referral completion. HIT interventions, such as electronic medical records and mobile technology in health tools (mHealth), are a key strategy in improving patient encounter documentation, data capture, inter-provider communication, and follow-up for non-communicable disease management in LMICs [20–23]. Peer support approaches leverage the unique patient–patient interactions built on shared disease experiences to influence behavior change [24]. Peer navigators have been used to improve linkage and retention in care for patients with HIV, mental health, and cancer [25–31], and even facilitate referral completion for cancer care [32].

The *St*rengthening *Re*ferral *N*etworks for Mana*g*ement of Hyper*t*ension across the *H*ealth *S*ystem (STRENGTHS) study is a cluster randomized control trial that will evaluate the effectiveness and cost-effectiveness of a combined HIT and peer support intervention on referral completion, BP improvement, and CVD risk reduction in Kenya. The STRENGTHS study uses a transdisciplinary implementation research approach and is the first study to rigorously evaluate such an intervention to strengthen referral networks in LMICs.

## Methods/design

### Setting

The Academic Model Providing Access to Healthcare (AMPATH) program is an academic global health partnership between Moi Teaching and Referral Hospital (MTRH), Moi University College of Health Sciences, and a consortium of North American universities led by Indiana University [33–35]. In recognition of the growing non-communicable disease (NCD) burden in LMICs [36], AMPATH has established a Chronic Disease Management (CDM) Program in collaboration with the Ministry of Health (MOH). The CDM Program has enrolled over 15,000 patients with hypertension at 69 facilities spanning all levels of the health system, alongside system-wide approaches to task redistribution, use of mHealth and electronic decision support, and ensuring a reliable supply of medicines for hypertension and other NCDs [37–39]. At MTRH, there is an inpatient cardiac critical care unit, a specialist outpatient cardiology clinic, and advanced cardiac diagnostic services [40]. It is within this clinical infrastructure that we plan to develop and test our intervention.

**Fig. 1:**
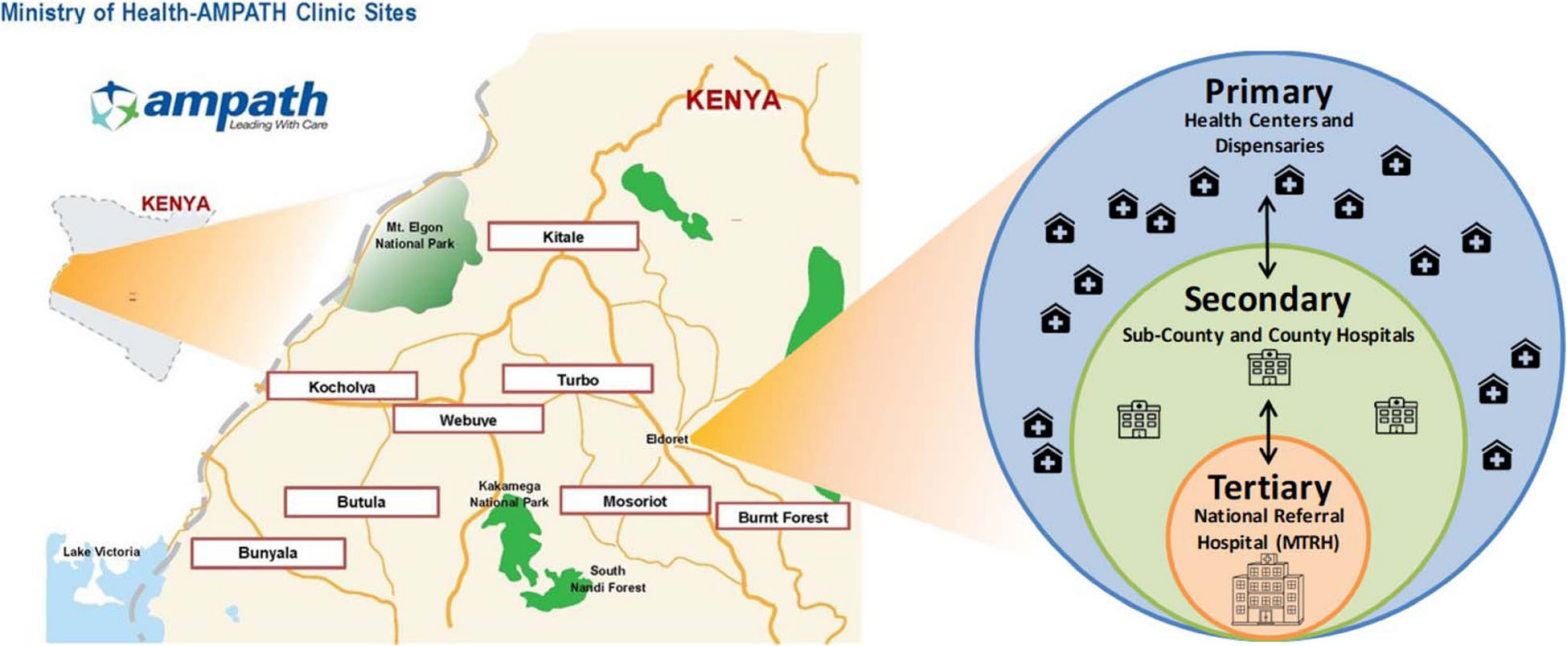
AMPATH clinic sites and referral network clusters; Health system levels

We will conduct this study within the AMPATH CDM program’s catchment area across eight geographically separate referral networks (Fig. 1). The Kenyan public-sector health system functionally coalesces into the three traditional health system levels—primary, secondary, and tertiary—and each referral network in our catchment area is centered around a secondary-level health facility: Kitale, Webuye, Kocholya, Turbo, Mosoriot, Burnt Forest, Bunyala, and Butula (Fig. 1). Each of these serves as the link between the common tertiary-level center, MTRH, staffed by specialists and sub-specialists, and several primary-level health facilities that feed into it, staffed by clinical officers (mid-level providers) and nurses (Fig. 1).

### Aims

The purpose of this study is to use a transdisciplinary, implementation science research approach to strengthen referral networks to improve hypertension control and reduce CVD risk. Our central hypothesis is that HIT integrated with peer support will be effective and cost-effective in strengthening referral networks, improving BP control, and reducing CVD risk among patients with hypertension in western Kenya.

**Fig. 2:**
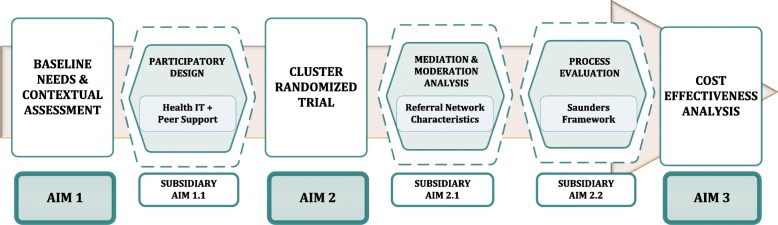
STRENGTHS study aims

The aims of the STRENGTHS study (Fig. 2) are to:

1. Conduct a baseline needs and contextual assessment, using a mixed-methods approach, for implementing and integrating HIT and peer support to strengthen referral networks for hypertension control. We will use these data to develop a contextually and culturally appropriate intervention using a participatory, iterative design process.
2. Evaluate the effectiveness of HIT and peer support for hypertension control by conducting a two-arm cluster randomized trial comparing: 1) usual care for referrals vs 2) referral networks strengthened with an integrated HIT and peer support intervention. The primary outcome will be one-year change in systolic blood pressure (SBP). The key secondary clinical outcome will be CVD risk reduction, and the key secondary implementation outcomes will include referral process metrics such as referral appropriateness and completion rates. We will conduct mediation and moderation analyses to evaluate the interaction between referral network characteristics and intervention outcomes. We will also conduct a process evaluation [41].
3. Evaluate the incremental cost-effectiveness of HIT and peer support, in terms of costs per unit decrease in SBP, per percentage change in CVD risk score, and per disability-adjusted life year (DALY) saved.

### Implementation framework

**Fig. 3:**
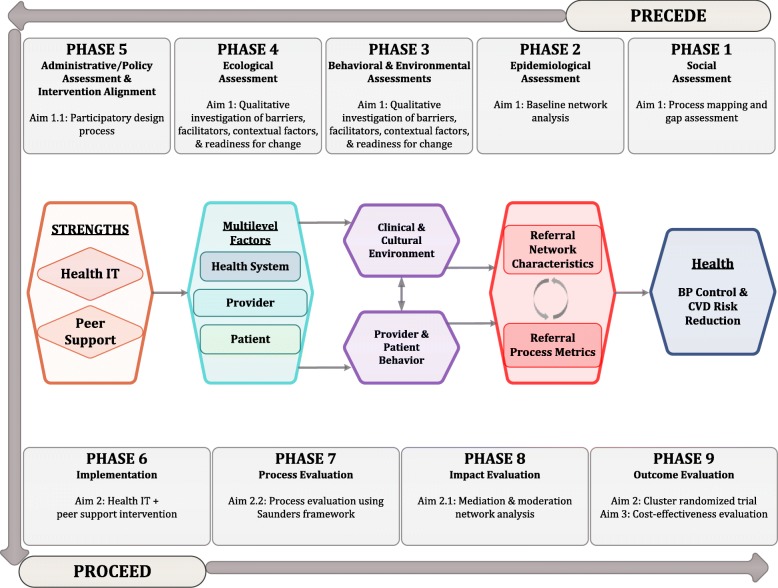
PRECEDE-PROCEED implementation framework and conceptual model of change

We are using the PRECEDE-PROCEED implementation science conceptual framework to help guide our study (Fig. 3) [42, 43]. We chose the PRECEDE-PROCEED framework because of its participatory nature, multi-pronged evaluation approach, and explicit assessment of the multi-level factors that may affect the uptake and success of our intervention. PRECEDE-PROCEED has also been successful in developing interventions to promote cardiovascular health in low-income populations [44].

### Conceptual model

Both HIT and peer support may strengthen referral networks, improve BP control, and reduce CVD risk through multiple mechanisms. HIT may address barriers to hypertension control at the provider and health system levels, by improving data quality, integrating and coordinating care across levels of the health system [45], and improving communication between providers [11]. The clinical decision support programmed into the HIT platform may also directly lead to improvements in referral appropriateness. Peer navigators may address barriers at the patient and health system levels, by helping patients navigate health system complexity and overcome barriers to health seeking behavior [46]. By addressing these multi-level factors, we hypothesize that our intervention will change both the clinical environment as well as patient and provider behaviors, which, in turn, will lead to improvements in referral network characteristics and referral process metrics. This has the potential to improve the coordination of care for patients with hypertension across the health system, ultimately reducing BP and CVD risk.

### Baseline needs and contextual assessment and participatory design process

We will use a mixed-methods approach to conduct a baseline needs and contextual assessment for implementing an integrated HIT and peer support intervention for strengthening referral networks for hypertension control. This will include: 1) observational process mapping and gap assessment; 2) baseline referral network analysis; and 3) a combination of qualitative research methods to identify facilitators, barriers, contextual factors, and readiness for change that may impact implementation of the integrated STRENGTHS intervention.

Observational process mapping, a technique adapted from Lean Six Sigma, will be conducted in order to determine patient and data flow throughout the referral network at each level of the health system [47, 48]. We will use a standardized checklist, adapted from the Kenya Health Sector Referral Strategy [49], to observe patient and data movement throughout the system, as well as detail the personnel, supplies, equipment, and logistics necessary for the referral network to be functional. To augment this, clinical staff and patients will be recruited by purposive and snowball sampling for semi-structured interviews in order to describe all the steps in the referral process, understand associated costs and how this affects referrals, clarify staff roles and responsibilities, delineate data sources and mechanisms for communication, and elicit perspectives on challenges and opportunities for improving referral networks [47, 48].

A baseline referral network analysis will be conducted by recruiting all AMPATH clinicians at every facility participating in the trial to complete a baseline survey of referral preferences and patterns. We will ask clinicians to indicate other clinicians who they go to for advice, who they discuss clinical issues with, and who they refer patients to for hypertension care. We will also analyze actual clinician-to-clinician referral patterns measured from the medical record. This will form the basis of the mediation and moderation analysis we will conduct once the trial is completed.

Qualitative methods will involve a combination of focus group discussions (FGDs), key informant interviews, and community *mabaraza*, conducted at each level of the health system within each of the six referral network clusters. *Mabaraza* are traditional community assemblies in East African culture and have been used as a novel methodology in community-based participatory research [50, 51]. We will use purposive sampling to recruit patients and clinical staff for the FGDs, and clinic leaders and health system administrators for the key informant interviews. For the mabaraza and FGDs, discussions will be allowed to deviate from the initial guiding discussion topics if additional relevant issues emerge, and participatory techniques will be used to elicit emotional elements and encourage group interaction. Thematic content analysis will be performed, using both deductive (a priori) and inductive (emerging) codes. We will also assess inter-coder reliability, and a kappa-statistic will be calculated.

After the baseline needs and contextual assessment is complete, we will use these data to inform a participatory, iterative design process to fully develop the integrated HIT and peer support STRENGTHS intervention [52–54] A “design team” including a facilitator, research staff, clinical staff, informatics staff, peer navigators, and patients will be formed. The design process will involve three phases: brainstorming, conceptualization, and creation. This design process will use the findings from the baseline needs and contextual assessment alongside input from this diverse group of stakeholders to inform the detailed development of the integrated HIT and peer support intervention protocol. Once the intervention model is developed, we will conduct additional FGDs to assess acceptability [55], as well as a 3-month pilot of the intervention to evaluate feasibility [56].

### Cluster randomized trial

**Fig. 4:**
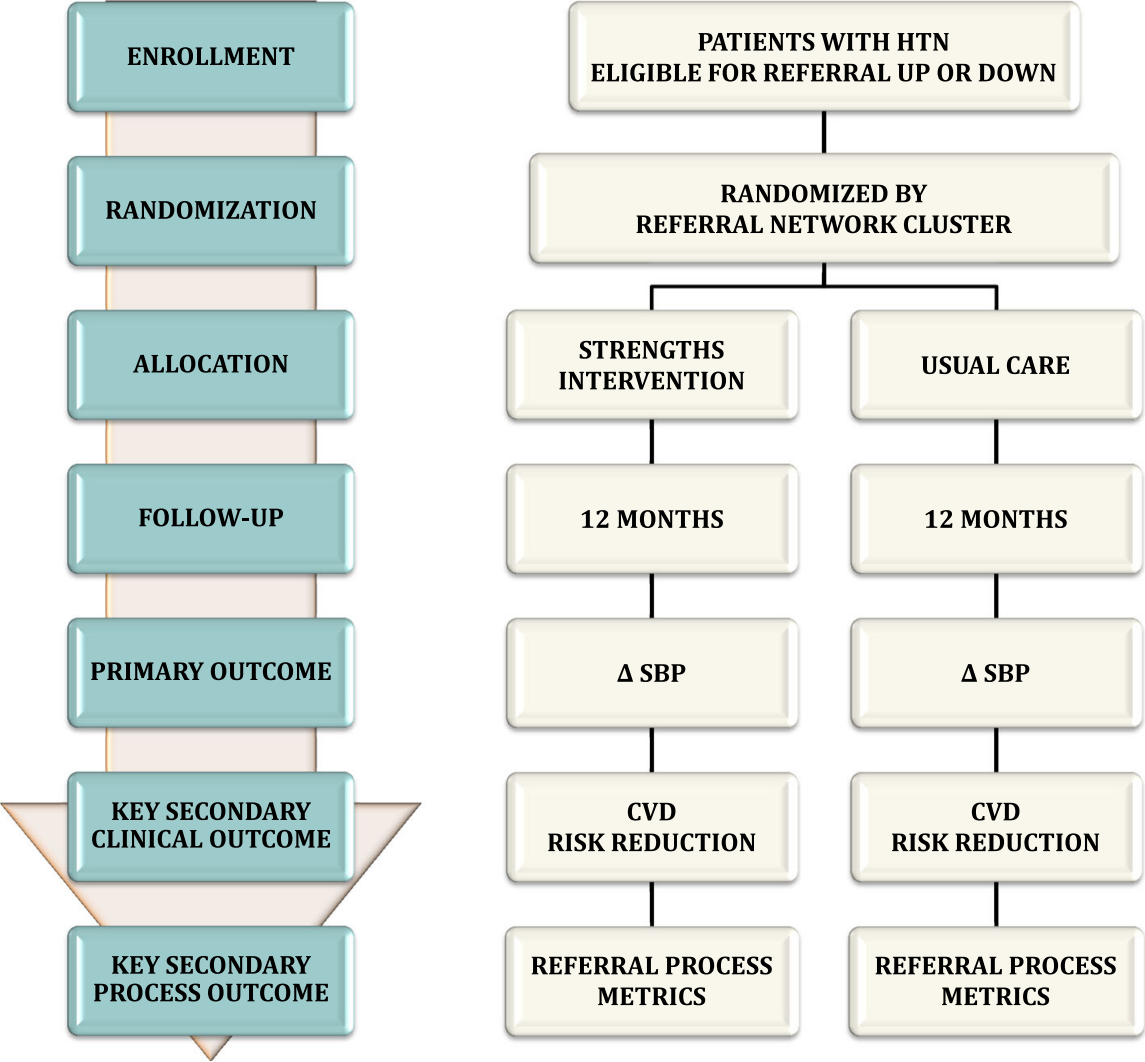
Flow of cluster randomized trial

**Fig. 5:**
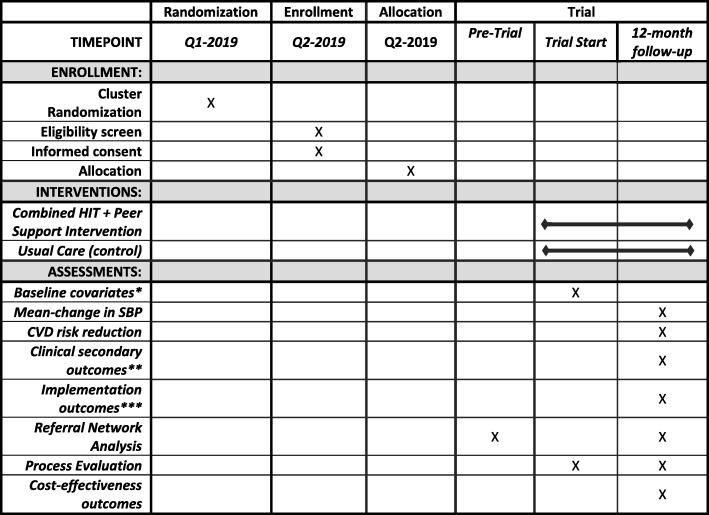
STRENGTHS SPIRIT figure

We will conduct a two-arm cluster randomized trial comparing usual care and referral networks strengthened with an integrated HIT and peer support intervention. The primary outcome measure will be one-year change in SBP and a key secondary outcome will be change in CVD risk score. There will be a total of eight clusters in the trial, four in each arm, with each cluster in a geographically distinct referral network within the overall AMPATH CDM program. First, each referral network cluster will be stratified by size of the health facility at the secondary level. Then cluster randomization will be done within each strata. Cluster randomization will be done using a computerized random number generator in a 1:1 allocation sequence between intervention and control groups. One of the investigators, separate from other research or clinical staff, will generate the allocation sequence and complete randomization. Study staff will then enroll participants in the trial. The trial flow chart is shown in Fig. 4 and the SPIRIT figure is shown in Fig. 5. The SPIRIT checklist is provided in Additional file 1.

### Study participants

Adult patients ≥ 18 years who are enrolled in AMPATH’s CDM program with hypertension at any level of care, and who meet criteria for referral up or down the referral network, will be eligible for inclusion in the trial. Individual informed consent will be obtained by study staff. Criteria for “referral up” the network is defined as patients with hypertension who remain uncontrolled (SBP ≥ 140 or diastolic blood pressure (DBP) ≥ 90) on three or more anti-hypertensive medications, who have signs or symptoms of end-organ damage, or who have suspected secondary causes of hypertension. Criteria for “referral down” the network is defined as controlled BP (SBP < 140 and DBP < 90) for three or more consecutive visits and no evidence of new end-organ damage. Exclusion criteria include acute illness requiring immediate medical attention, terminal illness, and inability to provide informed consent.

### Integrated HIT and peer support intervention

#### Usual care

For patients requiring referral up the network, the referring clinician writes a referral letter on a blank referral form. This referral letter is then given to the patient, who is responsible for presenting it to the receiving facility and arranging the referral visit. For patients requiring referral down the network, a counter-referral letter, and/or copies of clinical records, are given to the patient to take back to their primary facility, although in reality this is inconsistent.

#### HIT plus peer support referral intervention

**Fig. 6:**
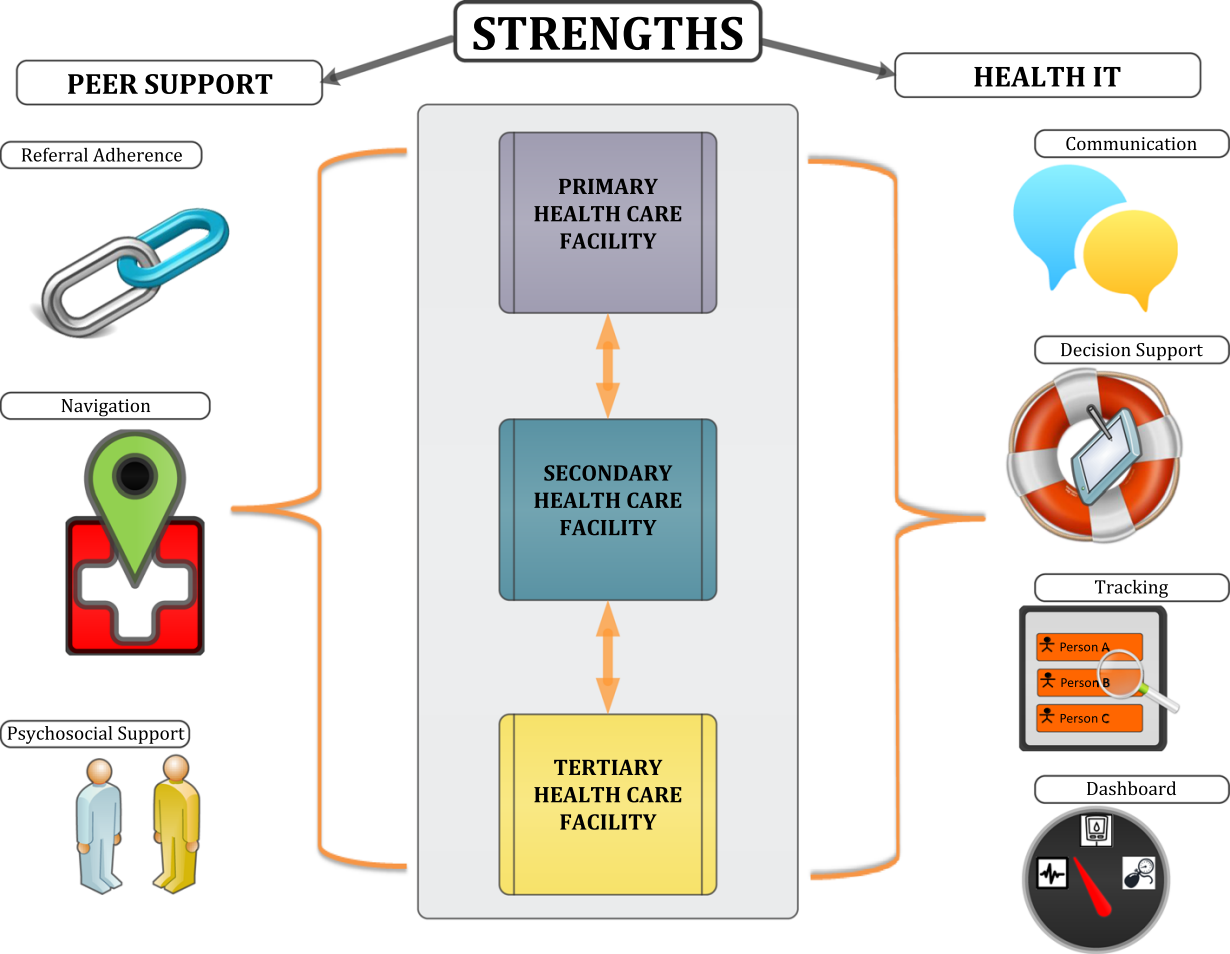
STRENGTHS intervention

Those randomized to the intervention group will receive clinical care for hypertension in the same manner as those randomized to the usual care group. Furthermore, refusal to participate in the trial or withdrawal from the trial will not affect clinical care provided. Reasons for referral will also be the same in the two groups. The change in referral process is the intervention (Fig. 6).

#### Health information technology

AMPATH uses the AMPATH Medical Record System (AMRS), a customized version of OpenMRS [22] that is centrally hosted and accessed by tablet via the Internet. Our HIT intervention will augment AMRS to support a referral system in four ways: 1) communication—facilitate data sharing by all providers and peer navigators across all levels of the health system; 2) decision support—provide clinical decision support to facilitate appropriate patient referrals; 3) tracking—generation and sharing of real-time patient referral lists; and 4) dashboards—create a platform for monitoring key referral process metrics. The system will prompt for referral based on clinical algorithms built into the clinical decision support, but also will allow the provider to initiate a referral based on his/her clinical judgment. Providers and peer-navigators will access the referral dashboard for a clinic to review patient referrals and track the status of referred patients. Key referral process metrics, such as referral wait times and completion rates, will be available in real-time for providers and peer navigators to monitor and act upon. Additional functionality of the HIT platform will be developed from the participatory design process described above.

#### Peer support

The peer support component of the intervention will involve peer navigators at each level of the referral network—primary, secondary, and tertiary. Peer navigators are patients within the AMPATH CDM program who have well-controlled hypertension; they have the shared experience of living with hypertension and navigating the same health system as the patients enrolled in the trial. The peer support intervention has three main functions: 1) referral adherence—enhance communication between clinicians and patients to improve referral adherence; 2) navigation—help patients navigate the complex health system [30]; and 3) psychosocial support—help patients overcome barriers to health seeking behavior. Referral adherence support will help enhance communication about the referral between the provider and patient by having a peer navigator meet with patients to review referral rationale and logistics. The second major role of the peer navigator is health system navigation, especially at the tertiary level. Here, the peer navigator can personally receive a referred patient and walk them through registration, scheduling, triage, and diagnostic work-up in order to streamline the referral process and ensure patients are not lost in the complexity of the system [57]. Furthermore, peer navigators will meet with patients after the clinic visit to provide any follow-up navigation between the pharmacy, laboratory, or imaging. The third major role of the peer navigator is to provide psychosocial support, helping patients overcome individual-level barriers to health-seeking behaviors, drawing from the innate trust inherent in their shared disease experience [24].

#### Integrated HIT plus peer support

The peer navigators will be equipped with a HIT tool, as described above, so they can see the same data as the clinicians in order to track and follow referred patients appropriately. When a clinician at the primary level refers a patient to a higher level of care, the peer navigator covering that patient’s community catchment area will automatically be alerted via the HIT tool so they can contact and meet the patient and review referral logistics. The peer navigator will then complete a referral navigation form on the HIT tool, which will automatically trigger a notification to the clinician and peer navigator at the receiving facility. Communication between the peer navigators at each level of the health system will be automated via the HIT tool to ensure seamless communication and data-sharing. Peer navigators at all levels will be trained to use the HIT tools, understand the basics of clinical referral algorithms and processes, navigate the health system, and provide education, counseling, motivational interviewing, and psychosocial support. Prior to trial enrollment, all clinicians will be sensitized about the STRENGTHS trial and educated on the STRENGTHS intervention. This will be done to help standardize the intervention delivery across clusters as well as to improve adherence and fidelity to the intervention model.

### Outcomes

The primary outcome is one-year absolute mean change in SBP. The BP measurement for inclusion in the trial, and used in the primary outcome analysis, will be standardized and conducted by trained study staff. The key secondary clinical outcome is one-year change in overall CVD risk as measured by the QRISK2 score, which has been validated for calculating 10-year CVD event risk for Black Africans [58–66]. Treatment effect will be average change in SBP and QRISK2 score from baseline to month 12 and will be estimated using an intention-to-treat approach with appropriately specified regression models. Other clinical secondary outcomes will include mortality, hospital admissions, CVD complications, change in CVD risk factors and behaviors, and medication adherence [67]. The primary outcome and clinical secondary outcomes pertain to the individual participant level. We will also measure referral process metrics as key secondary implementation outcomes, including referral completion rates, median referral completion time, referral eligibility, referral indication, and referral appropriateness. Data will be collected on covariates we hypothesize may be related to our outcomes. These include patient demographics, socioeconomic and education status, clinical comorbidities, CVD risk factors, health behaviors, medication adherence, geographic location, referral level, provider level of training, and peer navigator and provider characteristics.

### Statistical power

We expect that each of the six referral networks will enroll at least 200 participants. Given the longstanding history of the AMPATH CDM program and robust clinical infrastructure in our catchment area, as well as previous success in recruiting participants for community- and clinical-based research related to hypertension and other chronic diseases, we anticipate being able to successfully enroll our needed number of participants. To augment this, we have sensitized all participating AMPATH CDM staff on the STRENGTHS trial, and conducted training of clinicians on the AMPATH CDM hypertension treatment and referral guidelines. Through our baseline needs and contextual assessment (Aim 1), clinic staff have become well versed on the eligibility criteria and enrollment procedures, which will also help with recruitment for the trial. During our pilot testing, if we encounter difficulty with recruitment or enrollment, then we will put additional strategies in place to overcome this. We estimate a 10-mmHg reduction in SBP in the intervention arm compared to the usual care arm [68]. For our power calculation, we set the type I and type II error rates at 5% and 20%, respectively. We account for within-cluster correlation using intra-class correlation (ICC) values ranging from 0.02 to 0.06 consistent with prior studies using SBP as the outcome [69]. We analyze scenarios over a range of minimum detectable differences (MDD) assuming a standard deviation (SD) for change in SBP of 15 from the literature, with SD ranges from 10 to 20 [69, 70]. Finally, we assume that up to 15% of enrolled participants will be lost to follow-up. Table 1 presents expected power assuming a standard deviation of 15 for the comparison across different combinations of ICC and minimum detectable differences (MDD). Over a wide range of plausible ICC, with standard deviation fixed at 15, we will be able to detect a mean difference in change in SBP consistent with the published literature [69, 70] and our preliminary data [71, 72] with power greater than 80%.

**Table 1:** Estimated power over a range of ICC and MDD values

MDD	ICC				
	0.02	0.03	0.04	0.05	0.06
4	36	27	22	19	17
6	65	51	42	36	32
8	87	74	64	56	50
10	96	89	82	74	67
12	99	97	92	87	82
14	99	99	98	95	91

### Analytical approach

We will use an analysis of covariance model with cluster effects to estimate and draw inference about treatment effects for both primary and key secondary outcomes. The model specification is:

$$ {\mathrm{y}}_{\mathrm{ij}}=\upalpha +{\upgamma}_{\mathrm{j}}+{\upbeta}_1{\mathrm{x}}_{\mathrm{ij}}+\uptheta {\mathrm{b}}_{\mathrm{ij}}+{\mathrm{e}}_{\mathrm{ij}} $$

where y_ij_ is SBP at the end of follow-up for the ith individual in the jth cluster, b_ij_ is baseline SBP, and the indicators x_ij_ represent intervention group membership, with usual care as the reference. Hence, β coefficient represents the difference in mean change in SBP between usual care and the intervention. The intercept is α and γ_j_ is the effect of the jth cluster. Cluster effects γ_j_ will initially be modeled as fixed effects, but we will use a mixed model structure and assign a normal distribution if the data support this assumption. We will compare key independent variables across the trial arms to ensure balance of the randomization process and adjust the model to estimate treatment effects as needed. For modeling QRISK2, we will use the transformation log(−log (QRISK2)) because the risk score ranges from 0% to 100% [73]. We will test the further hypotheses that changes in referral network characteristics and referral process metrics can mediate the effect of the intervention on CVD outcomes, and that baseline referral network characteristics can moderate the effect of the intervention on outcomes, by conducting a mediation and moderation analysis, respectively [74, 75]. Further details of the data management procedures and statistical methods, including approach to missing data, mediation and moderation analysis, and secondary outcomes, are available upon request from the authors.

### Process evaluation

We will use the Saunders Framework [41] to conduct a process evaluation of the integrated HIT and peer support intervention. This process evaluation will be undertaken in order to monitor program implementation and better understand the relationship between intervention components and health outcomes. The Saunders Framework provides a systematic approach for guiding the evaluation of key implementation process measures across the following domains: fidelity (quality), dose delivered (completeness), dose received (exposure and satisfaction), recruitment, reach (participation rate), and context. Mixed quantitative and qualitative methods will be used to collect data from study participants and clinical staff, as well as from study and clinical encounter forms. This process evaluation will generate both formative and summative data [76, 77].

A combination of quantitative and qualitative approaches will be taken, including observation forms, checklists, surveys, record review, focus group discussions, and semi-structured interviews. Study participants, clinical staff, and study and clinical encounter forms will be the sources of data. Descriptive statistics will be generated for quantitative data, and thematic content analysis will be performed for qualitative data. Formative data will be reported iteratively to the study team and staff throughout the trial, while summative data will be reported at the end of the trial.

### Cost-effectiveness analysis

We will evaluate the incremental cost-effectiveness of HIT and peer support in terms of costs per unit change in SBP, per percentage change in CVD risk score, and per DALY saved. For each intervention arm, costs and potential cost offsets from the societal and payer perspectives will be captured using previously validated cost questionnaires used in Kenya for hypertension trials [78–80]. Study participants will complete one costing instrument, capturing healthcare utilization and expenditures, work loss, and transportation costs. All measures of burden will be monetized and, if there are differences across arms, used to quantify cost offsets from intervention delivery. Research staff will complete an additional costing instrument in order to capture all relevant labor, materials, supplies, and contracted services costs for all intervention activities. Only net incremental costs, after removing sunk costs and after factoring in cost offsets, will be included as the numerator in the cost-effectiveness analysis as these are the relevant costs for decision makers. We will also identify which activities drive the overall costs, and how costs would change if specific activities are added or eliminated. Effectiveness in terms of SBP and CVD risk score will be defined as above. DALY improvements will be estimated using well-defined relationships between blood pressure reductions and CVD DALYs averted [81]. We will also conduct sensitivity analyses to gauge the influence of key assumptions on the resulting incremental cost-effectiveness ratio.

## Discussion

The burden of hypertension and CVD in LMICs is immense, requiring a coordinated health system response. Ineffective referral networks in LMICs contribute substantially to CVD-related morbidity and mortality. The STRENGTHS study has been designed to strengthen referral networks in order to improve BP control and reduce CVD risk. The STRENGTHS study offers several innovative elements with some key advantages. First, while HIT and peer support are proven interventions to improve coordination of care and health outcomes, their application in an integrated fashion to address multi-level factors in order to improve referral networks for BP control and CVD risk reduction in LMICs is novel. Second, we are using a participatory design process to ensure our intervention model is culturally appropriate, context-specific, and responsive to community needs and local health system realities. Third, we use PRECEDE-PROCEED, applying an implementation science theoretical framework to guide our study. Fourth, we are evaluating for the possible mediating and moderating influence of referral network characteristics on intervention outcomes, as well as including a rigorous cost effectiveness analysis. Fifth, we are a team of transdisciplinary investigators with expertise in cardiology, internal medicine, pharmaceutical care, health systems, economics, informatics, social network analysis, qualitative methods, biostatistics, and implementation science. Finally, we are layering this implementation research approach onto the existing clinical care system, maximizing the likelihood, if successful, for continued adoption and sustainability. While this research is situated within a particular health system in a single country focused on a single disease outcome, we anticipate that both the methodology and results can be applied to other settings.

Our study has some limitations and challenges. First, our baseline needs and contextual assessment is critical to designing our intervention model and implementing our trial, and there may be resistance by the community to participation. To mitigate this, we will hold sensitization sessions among clinic staff and patients before initiation, and we have been successful with this approach in previous studies in this setting [50, 51, 82]. Second, we are conducting a two-arm trial with a combined (HIT plus peer support) intervention group, rather than a four-arm trial studying each individual intervention component, plus a separate combined group. We will therefore not be able to determine which component of the intervention was responsible for the observed effects on the primary and secondary outcomes. We chose this approach because a four-arm trial, randomized at the level of the referral network cluster, would be difficult to power and logistically complex to implement in the context of the existing care program. Our process evaluation will help to elucidate which components of the intervention worked, and how they performed on key implementation measures. A third limitation is that blinding of the study participants and research staff is not possible due to the design of the intervention. This may lead to bias, although we do not anticipate this to be a major issue given integration into the existing care system and the geographically distinct clusters. Despite these limitations, we anticipate that the STRENGTHS study will generate important results and provide a scalable health systems intervention for chronic diseases globally.

## Trial status

The study-specific manual of operations and policies have been developed, and training of research and clinical care staff is being completed. The Data Safety and Monitoring Board has been formed. The baseline needs and contextual assessment of Aim 1 has been completed and data analysis is underway. Enrollment into the trial is planned to start in August 2019 and be completed by July 2010. This is study protocol version 6.0 and the version date is May 6, 2019.

## Additional file

Additional file 1: SPIRIT 2013 Checklist: Strengthening Referral Networks for Management of Hypertension Across the Health System (STRENGTHS) in western Kenya: a study protocol of a cluster randomized trial. (DOCX 54 kb)

## Data Availability

Upon completion of the trial, datasets used and/or analyzed during the current study are available from the corresponding author on reasonable request to the AMPATH Research Program Office.

## Abbreviations

AMPATH: Academic Model Providing Access to Healthcare
BP: Blood pressure
CDM: Chronic Disease Management Program
CVD: Cardiovascular disease
DALY: Disability adjusted life year
FGD: Focus group discussion
HIT: Health information technology
ICC: Intra-class correlation
LMICs: Low- and middle-income countries
MDD: Minimum detectable difference
mHealth: Mobile technology in health tools
MOH: Ministry of health
MTRH: Moi Teaching and Referral Hospital
NCD: Non-communicable disease
SBP: Systolic blood pressure
SD: Standard deviation
STRENGTHS: Strengthening Referral Networks for Management of Hypertension Across the Health System

## References

[1] GBD 2016 Risk Factors Collaborators. Global, regional, and national comparative risk assessment of 84 behavioural, environmental and occupational, and metabolic risks or clusters of risks, 1990–2016: a systematic analysis for the Global Burden of Disease Study 2016. Lancet (London, England). 2017;390(10100):1345–422. 10.1016/S0140-6736(17)32366-8 PMC5614451 28919119

[2] Jha V, Garcia-Garcia G, Iseki K, Li Z, Naicker S, Plattner B (2013). Chronic kidney disease: global dimension and perspectives. Lancet (London, England).

[3] Chow CK, Teo KK, Rangarajan S, Islam S, Gupta R, Avezum A (2013). Prevalence, awareness, treatment, and control of hypertension in rural and urban communities in high-, middle-, and low-income countries. JAMA.

[4] World Health Organisation. Global action plan for the prevention and control of noncommunicable diseases 2013-2020. Geneva: World Health Organisation; 2013.

[5] Jamison DT, Breman JG, Measham AR, Alleyne G, Claeson M, Evans DB (2006). Disease control priorities in developing countries.

[6] WHO (2000). The world health report 2000 - Health systems: improving performance.

[7] Ilboudo TP, Chou YJ, Huang N (2012). Compliance with referral for curative care in rural Burkina Faso. Health Policy Plan.

[8] Levitt NS, Puoane T, Denman CA, Abrahams-Gessel S, Surka S, Mendoza C (2015). Referral outcomes of individuals identified at high risk of cardiovascular disease by community health workers in Bangladesh, Guatemala, Mexico, and South Africa. Glob Health Action.

[9] Nanyonjo A, Bagorogoza B, Kasteng F, Ayebale G, Makumbi F, Tomson G (2015). Estimating the cost of referral and willingness to pay for referral to higher-level health facilities: a case series study from an integrated community case management programme in Uganda. BMC Health Serv Res.

[10] Uwemedimo OT, Arpadi SM, Chhagan MK, Kauchali S, Craib MH, Bah F (2014). Compliance with referrals for non-acute child health conditions: evidence from the longitudinal ASENZE study in KwaZulu Natal, South Africa. BMC Health Serv Res.

[11] Bahous MC, Shadmi E (2016). Health information exchange and information gaps in referrals to a pediatric emergency department. Int J Med Inform.

[12] Kowalewski M, Jahn A, Kimatta SS (2000). Why do at-risk mothers fail to reach referral level? Barriers beyond distance and cost. Afr J Reprod Health.

[13] Orimadegun AE, Akinbami FO, Akinsola AK, Okereke JO (2008). Contents of referral letters to the children emergency unit of a teaching hospital, southwest of Nigeria. Pediatr Emerg Care.

[14] Pembe AB, Carlstedt A, Urassa DP, Lindmark G, Nystrom L, Darj E (2010). Effectiveness of maternal referral system in a rural setting: a case study from Rufiji district, Tanzania. BMC Health Serv Res.

[15] Pembe AB, Urassa DP, Darj E, Carlsted A, Olsson P (2008). Qualitative study on maternal referrals in rural Tanzania: decision making and acceptance of referral advice. Afr J Reprod Health.

[16] Grace SL, Chessex C, Arthur H, Chan S, Cyr C, Dafoe W (2011). Systematizing inpatient referral to cardiac rehabilitation 2010: Canadian Association of Cardiac Rehabilitation and Canadian Cardiovascular Society joint position paper endorsed by the Cardiac Care Network of Ontario. Can J Cardiol.

[17] Grace SL, Leung YW, Reid R, Oh P, Wu G, Alter DA (2012). The role of systematic inpatient cardiac rehabilitation referral in increasing equitable access and utilization. J Cardiopulm Rehabil Prev.

[18] Burtle D, Welfare W, Elden S, Mamvura C, Vandelanotte J, Petherick E (2012). Introduction and evaluation of a ‘pre-ART care’ service in Swaziland: an operational research study. BMJ Open.

[19] Youngleson MS, Nkurunziza P, Jennings K, Arendse J, Mate KS, Barker P (2010). Improving a mother to child HIV transmission programme through health system redesign: quality improvement, protocol adjustment and resource addition. PLoS One.

[20] Bloomfield GS, Vedanthan R, Vasudevan L, Kithei A, Were M, Velazquez EJ (2014). Mobile health for non-communicable diseases in Sub-Saharan Africa: a systematic review of the literature and strategic framework for research. Glob Health.

[21] Braitstein P, Einterz RM, Sidle JE, Kimaiyo S, Tierney W (2009). “Talkin’ about a revolution”: How electronic health records can facilitate the scale-up of HIV care and treatment and catalyze primary care in resource-constrained settings. J Acquir Immune Defic Syndr (1999).

[22] Mamlin BW, Biondich PG, Wolfe BA, Fraser H, Jazayeri D, Allen C (2006). Cooking up an open source EMR for developing countries: OpenMRS – a recipe for successful collaboration. AMIA Ann Symp Proc.

[23] Tierney WM, Rotich JK, Hannan TJ, Siika AM, Biondich PG, Mamlin BW (2007). The AMPATH medical record system: creating, implementing, and sustaining an electronic medical record system to support HIV/AIDS care in western Kenya. Stud Health Technol Inform.

[24] Funnell MM (2010). Peer-based behavioural strategies to improve chronic disease self-management and clinical outcomes: evidence, logistics, evaluation considerations and needs for future research. Fam Pract.

[25] Gabitova G, Burke NJ (2014). Improving healthcare empowerment through breast cancer patient navigation: a mixed methods evaluation in a safety-net setting. BMC Health Serv Res.

[26] Hatcher AM, Turan JM, Leslie HH, Kanya LW, Kwena Z, Johnson MO (2012). Predictors of linkage to care following community-based HIV counseling and testing in rural Kenya. AIDS Behav.

[27] Kelly E, Fulginiti A, Pahwa R, Tallen L, Duan L, Brekke JS (2014). A pilot test of a peer navigator intervention for improving the health of individuals with serious mental illness. Community Ment Health J.

[28] Maxwell AE, Jo AM, Crespi CM, Sudan M, Bastani R (2010). Peer navigation improves diagnostic follow-up after breast cancer screening among Korean American women: results of a randomized trial. Cancer Causes Control.

[29] Okeke NL, Ostermann J, Thielman NM (2014). Enhancing linkage and retention in HIV care: a review of interventions for highly resourced and resource-poor settings. Curr HIV/AIDS Rep.

[30] Park PH, Wambui CK, Atieno S, Egger JR, Misoi L, Nyabundi JS (2015). Improving diabetes management and cardiovascular risk factors through peer-led self-management support groups in western Kenya. Diabetes Care.

[31] Pastakia SD, Manyara SM, Vedanthan R, Kamano JH, Menya D, Andama B (2017). Impact of Bridging Income Generation with Group Integrated Care (BIGPIC) on hypertension and diabetes in rural western Kenya. J Gen Intern Med.

[32] Roland KB, Milliken EL, Rohan EA, DeGroff A, White S, Melillo S (2017). Use of community health workers and patient navigators to improve cancer outcomes among patients served by federally qualified health centers: A systematic literature review. Health Equity.

[33] Bloomfield GS, Kimaiyo S, Carter EJ, Binanay C, Corey GR, Einterz RM (2011). Chronic noncommunicable cardiovascular and pulmonary disease in sub-Saharan Africa: an academic model for countering the epidemic. Am Heart J.

[34] Einterz RM, Kimaiyo S, Mengech HN, Khwa-Otsyula BO, Esamai F, Quigley F (2007). Responding to the HIV pandemic: the power of an academic medical partnership. Acad Med.

[35] Mercer T, Gardner A, Andama B, Chesoli C, Christoffersen-Deb A, Dick J (2018). Leveraging the power of partnerships: spreading the vision for a population health care delivery model in western Kenya. Glob Health.

[36] GBD 2016 Risk Factors Collaborators. Global, regional, and national comparative risk assessment of 79 behavioural, environmental and occupational, and metabolic risks or clusters of risks, 1990–2015: a systematic analysis for the Global Burden of Disease Study 2015. Lancet (London, England). 2016;388(10053):1659–724. 10.1016/S0140-6736(16)31679-8 PMC5388856 27733284

[37] Manji I, Manyara SM, Jakait B, Ogallo W, Hagedorn IC, Lukas S (2016). The Revolving Fund pharmacy model: Backing up the Ministry of Health supply chain in western Kenya. Int J Pharm Pract.

[38] Pastakia S, Manji I, Mercer T (2015). Noncommunicable diseases and essential medicines. Health Aff.

[39] Vedanthan R, Kamano JH, Bloomfield GS, Manji I, Pastakia S, Kimaiyo SN (2015). Engaging the entire care cascade in western Kenya: A model to achieve the cardiovascular disease secondary prevention roadmap goals. Glob Heart.

[40] Binanay CA, Akwanalo CO, Aruasa W, Barasa FA, Corey GR, Crowe S (2015). Building sustainable capacity for cardiovascular care at a public hospital in western Kenya. J Am Coll Cardiol.

[41] Saunders RP, Evans MH, Joshi P (2005). Developing a process-evaluation plan for assessing health promotion program implementation: a how-to guide. Health Promot Pract.

[42] Green LW, Kreuter MW (2005). Health program planning: An educational and ecological approach.

[43] Community Tool Box [Available from: http://ctb.ku.edu/en/table-contents/overview/other-models-promoting-community-health-and-development/preceder-proceder/main. Accessed 5 Sept 2019.

[44] Paradis G, O'Loughlin J, Elliott M, Masson P, Renaud L, Sacks-Silver G (1995). Coeur en sante St-Henri--a heart health promotion programme in a low income, low education neighbourhood in Montreal, Canada: theoretical model and early field experience. J Epidemiol Community Health.

[45] Murray SF, Pearson SC (2006). Maternity referral systems in developing countries: current knowledge and future research needs. Soc Sci Med (1982).

[46] Raut A, Thapa P, Citrin D, Schwarz R, Gauchan B, Bista D (2015). Design and implementation of a patient navigation system in rural Nepal: Improving patient experience in resource-constrained settings. Healthcare (Amsterdam, Netherlands).

[47] Carter PM, Desmond JS, Akanbobnaab C, Oteng RA, Rominski SD, Barsan WG (2012). Optimizing clinical operations as part of a global emergency medicine initiative in Kumasi, Ghana: application of Lean manufacturing principals to low-resource health systems. Acad Emerg Med Off J Soc Acad Emerg Med.

[48] Lin SY, Gavney D, Ishman SL, Cady-Reh J (2013). Use of lean sigma principles in a tertiary care otolaryngology clinic to improve efficiency. Laryngoscope.

[49] Ministry of Health. Kenya health sector referral strategy 2014-2018. [Available at: http://guidelines.health.go.ke:8000/media/Ministry-of-Health-Referral-Strategy1.pdf]. Accessed 5 Sept 2019.

[50] Naanyu V, Vedanthan R, Kamano JH, Rotich JK, Lagat KK, Kiptoo P (2016). Barriers influencing linkage to hypertension care in Kenya: qualitative analysis from the lark hypertension study. J Gen Intern Med.

[51] Naanyu V, Sidle JE, Frankel RM, Ayuku D, Nyandiko WM, Inui TS (2011). Rooting inquiry in tradition: the health baraza as a tool for social research in Kenya. Qual Health Res.

[52] Brown T, Katz B (2011). Change by Design. J Prod Innov Manag.

[53] Leavy B (2010). Design thinking – a new mental model of value innovation. Strateg Leadersh.

[54] Brown T, Wyatt J (2010). Design thinking for social innovation. Stanf Soc Innov Rev.

[55] Nastasi BK, Varjas K, Schensul SL, Tudor Silva K, Schensul JJ, Ratnayake P (2000). The Participatory Intervention Model: A framework for conceptualizing and promoting intervention acceptability. Sch Psychol Q.

[56] Brockhouse JW, Wadsworth JJ (2010). Vital steps: A cooperative feasibility study guide.

[57] Karwa R, Maina M, Mercer T, Njuguna B, Wachira J, Ngetich C (2017). Leveraging peer-based support to facilitate HIV care in Kenya. PLoS Med.

[58] Hippisley-Cox J, Coupland C, Vinogradova Y, Robson J, Minhas R, Sheikh A (2008). Predicting cardiovascular risk in England and Wales: prospective derivation and validation of QRISK2. BMJ (Clinical research ed).

[59] Hippisley-Cox J, Coupland C, Vinogradova Y, Robson J, May M, Brindle P (2007). Derivation and validation of QRISK, a new cardiovascular disease risk score for the United Kingdom: prospective open cohort study. BMJ (Clinical research ed).

[60] Hippisley-Cox J, Coupland C, Vinogradova Y, Robson J, Brindle P (2008). Performance of the QRISK cardiovascular risk prediction algorithm in an independent UK sample of patients from general practice: a validation study. Heart.

[61] Collins GS, Altman DG (2009). An independent external validation and evaluation of QRISK cardiovascular risk prediction: a prospective open cohort study. BMJ (Clinical research ed).

[62] Collins GS, Altman DG (2010). An independent and external validation of QRISK2 cardiovascular disease risk score: a prospective open cohort study. BMJ (Clinical research ed).

[63] Schofield P, Crichton N, Chen R (2012). Methods for assessing cardiovascular disease risk in a UK black population. Heart.

[64] Tillin T, Hughes AD, Whincup P, Mayet J, Sattar N, McKeigue PM (2014). Ethnicity and prediction of cardiovascular disease: performance of QRISK2 and Framingham scores in a U.K. tri-ethnic prospective cohort study (SABRE--Southall And Brent REvisited). Heart (British Cardiac Society).

[65] Hippisley-Cox J, Coupland C, Robson J, Brindle P (2014). QRISK2 validation by ethnic group. Heart (British Cardiac Society).

[66] Tillin T, Hughes AD, Whincup P, Mayet J, Sattar N, McKeigue PM (2014). QRISK2 validation by ethnic group. Heart (British Cardiac Society).

[67] Voils CI, Maciejewski ML, Hoyle RH, Reeve BB, Gallagher P, Bryson CL (2012). Initial validation of a self-report measure of the extent of and reasons for medication nonadherence. Med Care.

[68] Vedanthan R, Kamano JH, Naanyu V, et al. Optimizing linkage and retention to hypertension care in rural Kenya (LARK hypertension study): study protocol for a randomized controlled trial. Trials. 2014;15:143. 10.1186/1745-6215-15-143 PMC4113229 24767476

[69] Mendis S, Johnston SC, Fan W, Oladapo O, Cameron A, Faramawi MF (2010). Cardiovascular risk management and its impact on hypertension control in primary care in low-resource settings: a cluster-randomized trial. Bull World Health Organ.

[70] Kengne AP, Awah PK, Fezeu LL, Sobngwi E, Mbanya JC (2009). Primary health care for hypertension by nurses in rural and urban sub-Saharan Africa. J Clin Hypertens (Greenwich, Conn).

[71] Pastakia SD, Manyara SM, Kamano JH, Menya D, Andama B, Laktabai J (2014). Bridging Income Generation Through the Provision of Incentives for Care (BIGPIC).

[72] Manyara S (2014). An integrated solution to management of diabetes and hypertension in western Kenya.

[73] Bartlett MS (1947). The use of transformations. Biometrics..

[74] MacKinnon DP, Fairchild AJ, Fritz MS (2007). Mediation analysis. Annu Rev Psychol.

[75] Tingley D, Yamamoto T, Hirose K, Keele L, Imai K. Mediation: R Package for Causal Mediation Analysis. J Stat Softw. 2014;59(5):38.

[76] Devaney B, Rossi P (1997). Thinking through evaluation design options. Child Youth Serv Rev.

[77] Helitzer D, Yoon SJ, Wallerstein N, Dow y Garcia-Velarde L (2000). The role of process evaluation in the training of facilitators for an adolescent health education program. J School Health.

[78] Gold DT, McClung B (2006). Approaches to patient education: Emphasizing the long-term value of compliance and persistence. Am J Med.

[79] Josyula S, Taylor KK, Murphy BM, Rodas D, Kamath-Rayne BD (2015). Obstetric referrals from a rural clinic to a community hospital in Honduras. Midwifery.

[80] Keyserling TC, Sheridan SL, Draeger LB, Finkelstein EA, Gizlice Z, Kruger E (2014). A comparison of live counseling with a web-based lifestyle and medication intervention to reduce coronary heart disease risk: a randomized clinical trial. JAMA Intern Med.

[81] Jafar TH, Islam M, Bux R, Poulter N, Hatcher J, Chaturvedi N (2011). Cost-effectiveness of community-based strategies for blood pressure control in a low-income developing country: findings from a cluster-randomized, factorial-controlled trial. Circulation.

[82] Vedanthan R, Tuikong N, Kofler C, Blank E, Kamano JH, Naanyu V (2016). Barriers and facilitators to nurse management of hypertension: A qualitative analysis from western Kenya. Ethn Dis.

